# Generalist arbuscular mycorrhizal fungi dominated heavy metal polluted soils at two artisanal and small − scale gold mining sites in southeastern Ecuador

**DOI:** 10.1186/s12866-022-02748-y

**Published:** 2023-02-15

**Authors:** Juan Pablo Suárez, Paulo Herrera, Carolina Kalinhoff, Oscar Vivanco-Galván, Selvaraj Thangaswamy

**Affiliations:** 1 Departamento de Ciencias Biológicas y Agropecuarias, Universidad Técnica Particular de Loja, UTPL, Loja, Ecuador; 2 PROMETEO Project, Loja, Ecuador

**Keywords:** AMF communities, Heavy metals, AMF-OTUs, Glomeromycotina, 18S nrDNA

## Abstract

**Background:**

Artisanal and small-scale gold mining activities are producing contamination with heavy metals and metalloids (HMM) into soils and water worldwide. The HMM are considered as one of the major abiotic stresses due to their long-term persistence in soil. In this context, arbuscular mycorrhizal fungi (AMF) confer resistance to a variety of abiotic plant stressors including HMM. However, little is known regarding the diversity and composition of AMF communities in heavy metal polluted sites in Ecuador.

**Methods:**

In order to investigate the AMF diversity, root samples and associated soil of six plant species were collected from two sites polluted by heavy metals, located in Zamora-Chinchipe province, Ecuador. The AMF 18S nrDNA genetic region was analyzed and sequenced, and fungal OTUs were defined based on 99% sequence similarity. Results were contrasted with AMF communities from a natural forest and from reforestation sites located in the same province and with available sequences in GenBank.

**Results:**

The main pollutants in soils were Pb, Zn, Hg, Cd and Cu with concentrations exceeding the soil reference value for agricultural use. Molecular phylogeny and OTU delimitation showed 19 OTUs, the family Glomeraceae was the most OTU-rich followed by Archaeosporaceae, Acaulosporaceae, Ambisporaceae and Paraglomeraceae. Most of the OTUs (11 of 19) have been found at other locations worldwide, 14 OTUs were proven from nearby non-contaminated sites in Zamora-Chinchipe.

**Conclusion:**

Our study showed that there are no specialized OTUs at the studied HMM polluted sites, but rather generalists adapted to a wide variety of habitats. Their potential role in phytoremediation approaches remains to be investigated.

## Background

Soil pollution by heavy metals is an increasing environmental problem worldwide. One of the most important sources of soil contamination is metal mining operations [26, 33]. The heavy metals and metalloids (HMM) are considered as one of the major abiotic stresses due to their long-term persistence in soil, provoking changes in plant functional traits [37] and structure of microbial communities [54, 56], particularly in arbuscular mycorrhizal fungi (AMF) [14]. AMF are members of sub-phylum Glomeromycotina, a ubiquitous component of terrestrial ecosystems, with many species worldwide distributed [51] and forming a symbiotic association with around 80 percent of vascular plants [50]. Mycorrhizal symbiosis plays a crucial role to improve the uptake of nutrients by plants, particularly on nutrient poor soils [39], confers resistance to a variety of abiotic plant stressors such as drought, salinity [7], and can alleviate heavy metal toxicity to their host plants [15].

Currently, in many South American countries, especially in Ecuador, gold continues to be extracted and processed through artisanal and small**-**scale gold mining (ASGM) resulting in heavy metals contamination of soil and water [47] as well as serious socio-environmental conflicts [45]. The province of Zamora-Chinchipe, specifically Chinapintza district, which is in the Paquisha cantón in the southeast of Ecuador, is one of the most affected areas with intensive exploitation of gold (Au) [46], silver (Ag) and copper (Cu), with a high potential of soil contamination. In Zamora-Chinchipe, around 26.8% (282.998 ha) of the total surface is franchised to miner exploitation [43], in which Chinapintza locality is exploited since pre-colonial times [36]. As a consequence of ASGM, the heavy metals, lead (Pb), zinc (Zn), cadmium (Cd) and Cu were reported as serious soil pollutants in Chinapintza [11].

However, dominant plant species present in HMM contaminated sites are habitually colonized by AMF, which is an indicator of their central role in mitigating heavy metal stress in plants [34, 62]. Chamba et al. [11] analyzed the potential use of *Axonopus compressus*, *Erato polymnioides* and *Miconia zamorensis* for phytoremediation of metal-contaminated soils in Chinapintza. In addition, in the same site, seven AMF spore morphotypes from *Glomus*, *Acaulospora*, *Ambispora* and *Racocetra* were associated with *E. polymnioides* and *M. zamorensis*, plant species considered as mercury accumulators [12]. The existing information derived from spore morphology may not reflect the root-associated AMF community [58]. In this context, the molecular identity of the involved AMF species is lacking taking into consideration plant species growing at these metal contaminated sites.

Previous studies of AMF communities present in natural forest and reforestation sites in the same Zamora-Chinchipe province were conducted [22, 20], revealing a diverse AMF community dominated by Operational Taxonomic Units (OTUs) belonging to Glomerales, followed by Diversisporales and Archaeosporales. Native AMF species shows better performance in metal remediation compared with the non-native fungi [29] consequently, information on local AMF adapted to soil conditions is relevant for applications such as phytoremediation. As far as we know, there is no previous report on the molecular diversity of AMF in soils polluted with heavy metals in gold-mining areas in Ecuador, and their role in favoring plant growth under such conditions.

Studies on AMF diversity in response to heavy metal contamination have been carried out in temperate or Mediterranean countries with ancient histories of land use in mining or other industrial activities. Orphan mining site in southern France with very high Zn (97,333 ppm), and Pb (31,333 ppm), presented a higher incidence of Glomerales, in addition to Paraglomerales and Diversisporales [44]. Mine tailings in Qiandongshan region (China) with high Pb (5899), and Zn (812 ppm), sustained 28 AMF species, among which *Rhizophagus intraradices*, *Funneliformis mosseae*, and *Acaulospora* sp. were the most abundant [60]. In a mining core over 2,500 years old with very high Al (14,500 ppm), Cd (37 ppm), Fe (190,300 ppm), Pb (6,900 ppm), and Zn (12,000 ppm), Alguacil et al. [1] found 12 AMF sequence types, and *G. mosseae* was the least frequent species. Three species of spontaneous colonizer plants of polluted zones around Dabaoshan Mine in China with very high Pb, Zn, and Cu had six *Glomus* ecotypes in their roots, while two other plants had only *Kuklospora* and *Ambispora* [35]. The same pattern of dominance of Glomeraceae has also been observed in urban and peri-urban locations contaminated by heavy metals. In areas within the city of Montreal with high concentrations of Pb and Zn caused by old depositions (60 years) of industrial and demolition waste, *G. mosseae* was found as the dominant ribotype [19]. In sites affected for 50 years by a copper smelter in South Korea (with higher concentrations of As, Zn, Cd, Cu and Pb) the dominant AMF were *Funneliformis mosseae* and *Rhizophagus intraradices* [28]. On the other hand, in areas affected by an old battery factory in the city of Córdoba, Argentina, a decrease in the relative proportion of Glomeraceae was observed, and an increase in Paraglomeraceae in response to Pb [14]. The results of these studies indicate that the dominance of AMF species and community assembly changes depending on the type, concentration, and combinations of heavy metals present. In some cases, host plant identity also has a significant effect on the composition of AMF communities under metal stress [1, 35].

The main objective of the present work is to investigate the diversity of AMF associated with six frequent plant species growing in soils polluted with heavy metals in mining areas of Chinapintza and La Pangui (Zamora-Chinchipe, Ecuador), and to understand whether the AMF taxa found are local specialists or rather generalists adapted to a variety of habitats. Phytoremediation with indigenous AMF [4] is discussed as an alternative to remediate soils in the studied sites.

## Results and discussion

### Physico-chemical analyses of soil samples and presence of AMF colonization

The analyses of 9 soil samples from Chinapintza and 21 soil samples from La Pangui showed low pH values, low nutrient content and high heavy metal concentration (Tables 1, 2). The lowest pH value and the highest concentration of heavy metals were detected at Chinapintza site (Table 2). The main heavy metals found at both sites were Pb, Zn, Hg, Cd and Cu, all with concentrations exceeding the soil reference value for agricultural use [10]; Table 2). There was a wide variability in the concentrations of the heavy metals from the different samples, which was also observed by Chamba et al. [11]. In our study, we consider the same species as [11, 12], *Axonopus compressus*, *Erato polymnioides* and *Miconia zamorensis*, but in addition, *Medinilla* sp., *Colacasia* sp. and *Cyathea* sp.

**Table 1:** Physico-chemical parameters analysed from soil samples collected from Chinapintza and La Pangui sites of Zamora-Chinchipe, Ecuador

Name of site	pH	Levels of major elements in ppm				Levels of trace elements in m.eq / 100 ml						Level of Na in meq / 100 g soil
		NH4	P	S	Cl	K	Ca	Mg	Fe	Mn	B	
Chinapintza^*^	3.39 ± 0.56	47.75 ± 29.25	17.34 ± 7.66	116.00 ± 80.84	89.80 ± 16.20	0.21 ± 0.13	0.68 ± 0.28	0.53 ± 0.45	1473.80 ± 926.2	150.64 ± 44.36	0.66 ± 0.14	0.22 ± 0.02
La Pangui^**^	4.25 ± 2.21	75.44 ± 39.66	22.54 ± 10.46	168.01 ± 33.99	83.80 ± 17.20	0.19 ± 0.14	2.98 ± 1.02	0.68 ± 0.23	661.20 ± 406.80	67.13 ± 40.14	0.82 ± 0.12	0.28 ± 0.10

^*^Mean value of 9 data

^**^Mean value of 21 data

**Table 2:** Levels of heavy metals on soils recorded from Chinapintza and La Pangui sites of Zamora-Chinchipe, Ecuador

Name of site	Levels of heavy metals in ppm							
	Al	Cd	Cr	Cu	Pb	Zn	Hg	Au
Chinapintza^*^	9426.83 ± 2211.24	4.25 ± 9.73	17.43 ± 3.51	131.84 ± 117.89	1501.25 ± 915.31	886.16 ± 595.30	26.26 ± 13.03	15.99 ± 7.13
La Pangui^**^	7922.37 ± 3556.19	3.13 ± 2.19	13.02 ± 8.50	94.83 ± 60.44	560.53 ± 283.53	460.42 ± 390.55	17.33 ± 12.88	9.58 ± 6.39
Soil quality reference^***^		1.4 – 22.0	64.0 – 87.0	63.0 – 91.0	70.0 – 600.0	200.0 – 360.0	6.6 – 50.0	

^*^Mean value of 4 data

^**^Mean value of 6 data

^***^Canadian Council of Ministers of the Environment (2007). The lower level is a reference value for agricultural and higher level is for industrial use

Despite the adverse soil conditions, examined root samples in both sites were moderate to highly colonized by AMF (40–80%) and showed the usual characteristics of AMF such as arbuscules, coils, extra and intracellular hyphae and vesicles (data not shown). This finding is consistent with Chamba et al. [11] in the same area of Chinapintza that showed mycorrhizal colonization of up to 70% in *E. polymnioides*, *M. zamorensis* and *A. compressus* (70 ± 7, 60 ± 5 and 50 ± 5%) respectively. Long et al. [35] earlier reported moderate to high degree of mycorrhizal colonization in five plant species growing in acidic soils severely polluted with Zn, Pb, Cu, and Cd. The extent of AMF colonization can be interpreted as positive correlation to plant dependence on symbiosis [55], even more under extreme soil conditions. The lack of data on essential soil factors, such as measurements of cation exchange capacity (CEC), phosphorus, and organic matter, is certainly a limitation of this study. Alguacil et al. [1] observed an increase in the percentage of colonization by AMF and a decrease in the concentration of heavy metals in native plants growing in polluted soil with organic amendments, indicating an increase in the resistance to heavy metals stress. In other cases, immobilization of metals such as Pb and Cd in roots and stems increases plant tolerance to heavy metals in presence of AMF colonization [24, 52].

### Molecular phylogeny and OTU delimitation

**Fig. 1:**
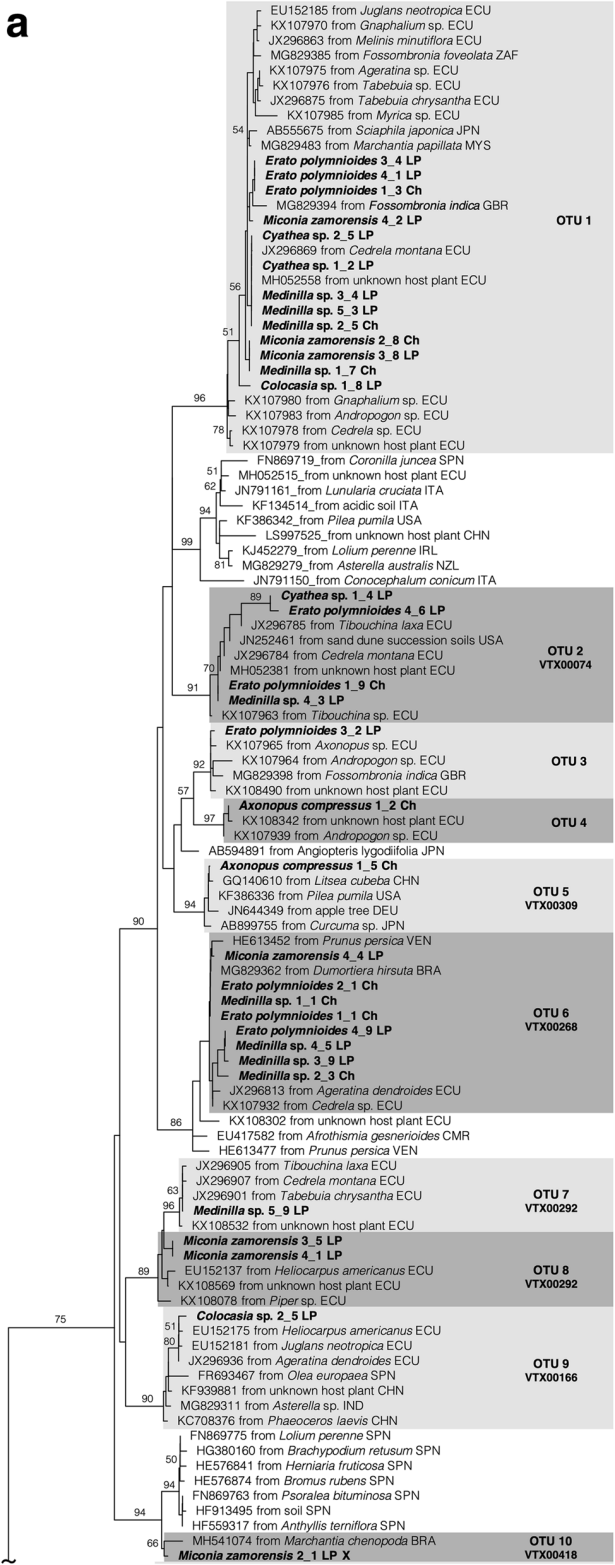

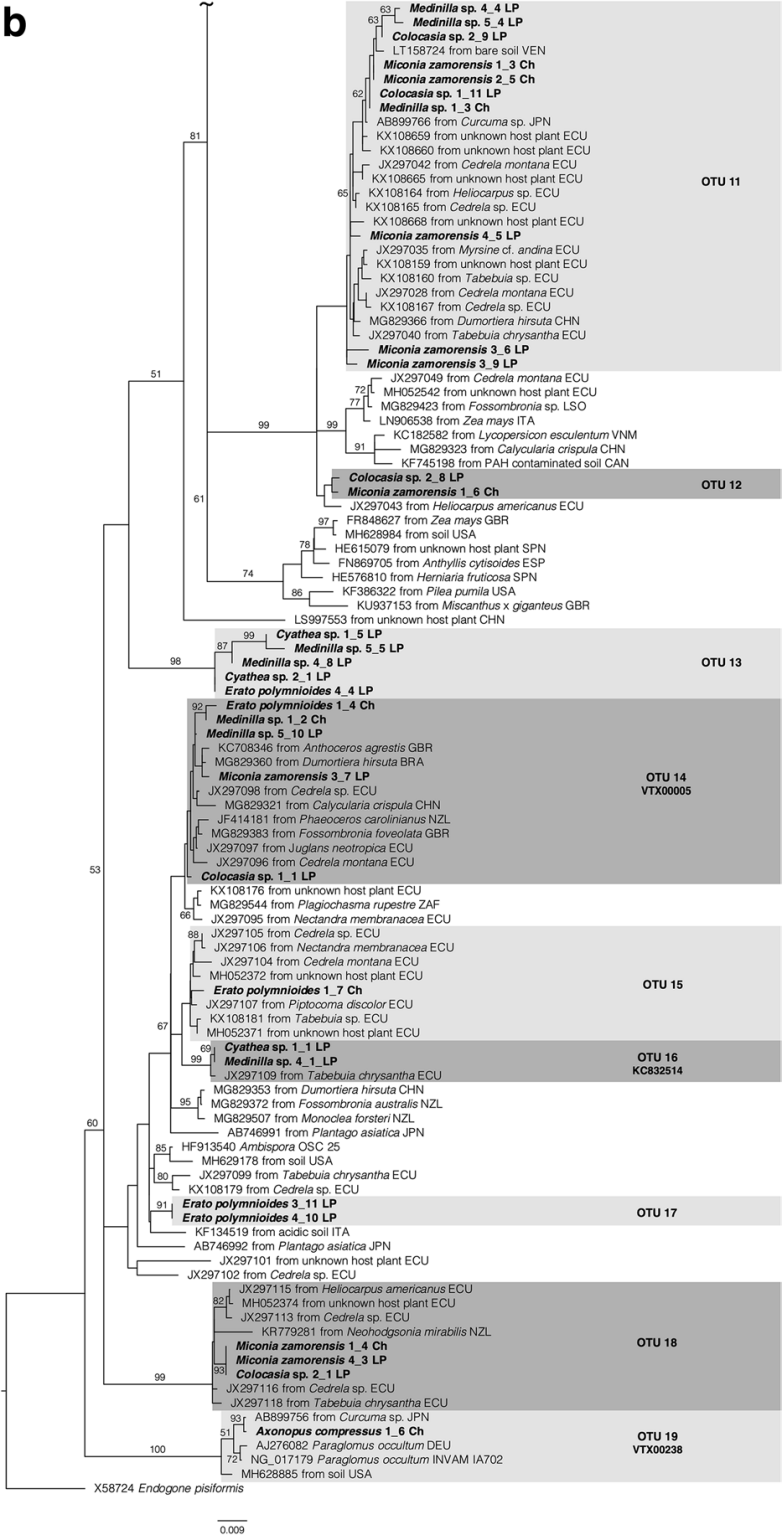
Phylogram inferred from a Maximum Likelihood (ML) analysis of partial 18S nrDNA sequences of Glomeromycotina associated with six plant species growing at heavy metal polluted soils in Chinapintza and La Pangui sites including sequences from the databases NCBI and MaarjAM with high similarity. The values that support the nodes correspond to Maximum Likelihood bootstrap. Only bootstrap values greater than 50% are shown. The tree was outgroup rooted with *Endogone pisiformis* X58724. OTUs are defined on a 99% similarity threshold. Sequences from this study are indicated by the species name, followed by individual number, clone number and location (Ch = Chinapintza and LP = La Pangui). Sequence provenances: BRA = Brazil, CAN = Canada, CHN = China, DEU = Germany, ECU = Ecuador, GBR = United Kingdom, IND = India, IRL = Ireland, ITA = Italy, JPN = Japan, LSO = Lesotho, MYS = Malaysia, NZL = New Zealand, SPN = Spain, USA = United States, VEN = Venezuela, VNM = Vietnam, ZAF = South Africa. Phylogenetic tree was divided into Fig. 1a and 1b

Successful PCR amplification was obtained from 18 plant samples in total, 7 samples from Chinapintza and 11 samples from La Pangui. After cloning 78 sequences of AMF were obtained, 64 sequences were grouped in 19 OTUs (Fig. 1 a, b and Table 3) and 14 sequences were singletons. The family Glomeraceae was the most diverse family displaying 52% (33) of all sequences and 53% (10) of all OTUs (OTU 1 to 10, Fig. 1 a). The Archaeosporaceae were represented with 15 sequences and 5 OTUs (OTU 13 to 17), Acaulosporaceae with 12 sequences and 2 OTUs (OTU 11 and OTU 12), Ambisporaceae with 3 sequences and 1 OTU (OTU 18) and Paraglomeraceae with 1 sequence and 1 OTU (OTU 19) (see Fig. 1 b). The most frequent OTU 1 Glomeraceae occurred in 72% of the samples (Table 3). The OTU 11 *Acaulospora* species is also frequent, occurring in 56% of the samples and a further Glomeraceae OTU 6 present in 44% of the samples. Many of the other OTUs were proven in smaller numbers, 8 only once.

**Table 3:** Frequency of AMF OTU at each plant individual recorded from samples collected from Chinapintza and La Pangui sites of Zamora-Chinchipe, Ecuador

Plan species	OTU^*^																			
	1	2	3	4	5	6	7	8	9	10	11	12	13	14	15	16	17	18	19	
*Axonopus compressus* 1				1	1														1	3
*Cyathea* sp 1	1	1											1			1				4
*Cyathea* sp 2	1												1							2
*Colacasia* sp 1	1										1			1						3
*Colacasia* sp 2									1		1	1						1		4
*Erato polymnioides* 1	1	1				1								1	1					5
*Erato polymnioides* 2						1														1
*Erato polymnioides* 3	1		1														1			3
*Erato polymnioides* 4	1	1				1							1				1			5
*Medinilla* sp 1	1					1					1			1						4
*Medinilla* sp 2	1					1														2
*Medinilla* sp 3	1					1														2
*Medinilla* sp 4		1				1					1		1			1				5
*Medinilla* sp 5	1						1				1		1	1						5
*Miconia sp* 1											1	1						1		3
*Miconia* sp 2	1									1	1									3
*Miconia* sp 3	1							1			2			1						5
*Miconia* sp 4	1					1		1			1							1		5
Total	13	4	1	1	1	8	1	2	1	1	10	2	5	5	1	2	2	3	1	64
La Pangui	1	1	1			1	1	1	1	1	1	1	1	1		1	1	1		15
Chinapintza	1	1		1	1	1					1	1		1	1			1	1	11

^*^OTU 1 to 10 correspond to Glomeraceae, OTU 11 and OTU 12 to Acaulosporaceae, OTU 13 to 17 Archaeosporaceae, OTU 18 Ambisporaceae and OTU 19 Paraglomeraceae

Most of the OTUs (14 of 19) were previously found in other locations in Ecuador, mostly in Zamora-Chinchipe province [20, 22], whereas 9 OTUs were previously found in Ecuador and several locations worldwide. For three OTUs (OTUs 12, 13 and 17) there were no proofs from other sites (Fig. 1 b and Table 4).

**Table 4:** List of sequences and previous recorded distribution corresponding at each OTU recorded from samples collected from Chinapintza and La Pangui sites of Zamora-Chinchipe, Ecuador

OTU	Freq	Plant species	Location		Family of Glomeromycotina	Previous recorded distribution
			LP	Ch		
1	13	*Colacasia* sp. 1_8	1		Glomeraceae	Ecuador^1,2^ and several locations worldwide (MYS^3^, JPN^4^, ZAF^3^, GBR^3^)
		*Medinilla* sp. 1_7		1		
		*Miconia zamorensis* 3_8	1			
		*Miconia zamorensis* 2_8		1		
		*Cyathea* sp. 2_5	1			
		*Medinilla* sp. 3_4	1			
		*Cyathea* sp. 1_2	1			
		*Medinilla* sp. 2_5		1		
		*Medinilla* sp. 5_3	1			
		*Erato polymnioides* 3_4	1			
		*Erato polymnioides* 4_1	1			
		*Erato polymnioides* 1_3		1		
		*Miconia zamorensis* 4_2	1			
2	4	*Erato polymnioides* 1_9		1	Glomeraceae	Ecuador^1,2^and USA^5^. Correspond to VTX00074 at MaarjAM database
		*Medinilla sp.* 4_3	1			
		*Cyathea sp.* 1_4	1			
		*Erato polymnioides* 4_6	1			
3	1	*Erato polymnioides* 3_2	1		Glomeraceae	Ecuador and GBR^3^
4	1	*Axonopus compressus* 1_2		1	Glomeraceae	Ecuador
5	1	*Axonopus compressus* 1_5		1	Glomeraceae	Several locations worldwide (CHN^6^, USA^7^, DEU, JPN) Correspond to VTX00309 at MaarjAM database
6	8	*Miconia zamorensis* 4_4	1		Glomeraceae	Ecuador^1^, VEN^8^ and BRA^3^. Correspond to VTX00268 at MaarjAM database
		*Erato polymnioides* 1_1		1		
		*Erato polymnioides* 2_1		1		
		*Medinilla* sp. 1_1		1		
		*Erato polymnioides* 4_9	1			
		*Medinilla* sp. 4_5	1			
		*Medinilla* sp. 3_9	1			
		*Medinilla* sp. 2_3		1		
7	1	*Medinilla* sp. 5_9	1		Glomeraceae	Ecuador^1^. Correspond to VTX00292 at MaarjAM database
8	2	*Miconia zamorensis* 3_5	1		Glomeraceae	Ecuador. Correspond to VTX00292 at MaarjAM database
		*Miconia zamorensis* 4_1	1			
9	1	*Colacasia* sp. 2_5	1		Glomeraceae	Ecuador^1^ and several locations worldwide (SPN^9^, CHN^10^, IND^3^). Correspond to VTX00166 at MaarjAM database
10	1	*Miconia zamorensis* 2_1_X	1		Glomeraceae	Brazil. Correspond to VTX00418 at MaarjAM database
11	10	*Medinilla* sp. 4_4	1		Acaulosporaceae	Ecuador^1^ and several locations worldwide (VEN, JPN, CHN^3^)
		*Medinilla* sp. 5_4	1			
		*Colacasia* sp. 2_9	1			
		*Miconia zamorensis* 1_3		1		
		*Miconia zamorensis* 2_5		1		
		*Colacasia* sp. 1_11	1			
		*Medinilla* sp. 1_3		1		
		*Miconia zamorensis* 3_6	1			
		*Miconia zamorensis* 3_9	1			
		*Miconia zamorensis* 4_5	1			
12	2	*Colacasia* sp. 2_8	1		Acaulosporaceae	
		*Miconia zamorensis* 1_6		1		
13	5	*Cyathea* sp. 1_5	1		Archaeosporaceae	
		*Medinilla* sp. 5_5	1			
		*Medinilla* sp. 4_8	1			
		*Cyathea* sp. 2_1	1			
		*Erato polymnioides* 4_4	1			
14	5	*Erato polymnioides* 1_4		1	Archaeosporaceae	Ecuador^1^ and several locations worldwide (GBR^3,10^, BRA^3^, CHN^3^, NZL^11^). Correspond to VTX00005 at MaarjAM database
		*Medinilla* sp. 1_2		1		
		*Medinilla* sp. 5_10	1			
		*Miconia zamorensis* 3_7	1			
		*Colacasia* sp. 1_1	1			
15	1	*Erato polymnioides* 1_7		1	Archaeosporaceae	Ecuador^1,2^
16	2	*Medinilla* sp. 4_1	1		Archaeosporaceae	Ecuador^1^
		*Cyathea* sp. 1_1	1			
17	2	*Erato polymnioides* 3_11	1		Archaeosporaceae	
		*Erato polymnioides* 4_10	1			
18	3	*Miconia zamorensis* 1_4		1	Ambisporaceae	Ecuador^1,2^ and NZL^12^
		*Miconia zamorensis* 4_3	1			
		*Colacasia* sp. 2_1	1			
19	1	*Axonopus compressus* 1_6		1	Paraglomeraceae	Ecuador and several locations worldwide (JPN, DEU^13^, USA). Correspond to CVTX00238 at MaarjAM database

[20],^1^ [21],^2^ [42],^3^ [59],^4^ [49],^5^ [35],^6^ [31],^7^ [3],^8^ [2],^9^ [13],^10^ [9],^11^ [16],^12^ [48]^13^

Sequences are indicated by the species name, followed by individual number and clone number. *Freq*  Frequency. Locations correspond to *Ch* Chinapintza and *LP* La Pangui. Sequence provenances as give in Fig. 1 ab: *BRA* Brazil, *CHN* China, *DEU* Germany, *ECU* Ecuador, *GBR* United Kingdom, *IND* India, *JPN* Japan, *MYS* Malaysia, *NZL* New Zealand, *SPN* Spain, *USA* United States, *VEN* Venezuela, *ZAF* South Africa

At La Pangui site 15 OTUs were detected, while at Chinapintza site 11 OTUs (Table 3). Seven OTUs were present at both sites, including all frequent ones. 3 to 8 OTUs per plant species and 1 to 5 OTUs per plant individual are present (Table 3 and Table 4). All plant species harbor Glomeraceae-OTUs and members of Archaeosporales with exception of *Axonopus compressus*, most of them also Acaulosporaceae (Table 3). The molecular analysis of AMF showed a species-rich community with 19 OTUs belonging to five different families: Glomeraceae, Acaulosporaceae, Archaeosporaceae, Ambisporaceae and Paraglomeraceae.

### Diversity of AMF fungi in heavy metal polluted mining areas

The highest concentration of heavy metals was detected at Chinapintza, with average values of Pb, Zn and Cu approximately double the value detected at La Pangui (Table 2). Previous studies have shown that an increase of HMM concentration decreases AMF richness [19, 60–62]. However, the observed difference in the number of OTUs between La Pangui and Chinapintza cannot attributed to differences in the concentration of heavy metals as the number of samples in both sites are not equivalent due to plant rarity.

Within Glomeraceae, OTU 1 was the most frequent followed by OTU 6, both present in the nearby sites of Zamora-Chinchipe, but also present elsewhere in the world. Our results are similar to those of previous studies showing the dominance of Glomeraceae in soils contaminated with heavy metals [8, 19, 28, 35, 44, 60]. In contrast, the most abundant AMF in several heavy metal contaminated soils *Rhizophagus intraradices* and *Funneliformis mosseae* [8, 19, 28, 60] were not found in our contaminated sites. Further studies are needed to determine whether host plant identity or site characteristics, such as climate or soil, have a significant effect on the composition of AMF communities.

In contrast to our results, in a recent study Faggioli et al. [14] found a rich AMF community dominated by members of Paraglomeraceae followed by Glomeraceae, in Pb-contaminated soils using an Illumina approach. In our study, together with the fact that 14 OTUs were previously found in other locations in Ecuador, it can be concluded that there are no specialists in heavy metal polluted sites, but generalists adapted to disturbed sites. However, the overall composition of the AMF-community of these heavy metal contaminated sites is similar to many other AMF-communities at family level: Glomeraceae dominates in terms of OTUs and frequencies, followed by Archaeosporaceae and Acaulosporaceae.

Zamora-Chinchipe, a place where artisanal and small-scale gold mining is a deeply rooted activity, provoking contamination for several decades, was object of study to investigate the diversity and composition of AMF communities associated with the roots of six plant species sampled from heavy metal polluted soils located in Chinapintza and La Pangui sites. Although several members of Glomeromycota are consider as cosmopolitan species [51], its local distribution is affected by several factors [23]. Some AM fungal taxa have only been reported in the highly contaminated areas, which could represent ecotypes adapted to this extreme environment [61, 62]. For phytoremediation of metal-contaminated soils, the use of indigenous fungi is recommended considering that they are adapted to particular abiotic and stressful conditions [41]. The potential role in phytoremediation approaches of the dominant fungi detected in our study remains to be investigated.

## Conclusion

We investigate the AMF diversity and associated soil of six plant species growing at two sites polluted by heavy metals. Overall results showed that there are no specialized OTUs at the studied HMM polluted sites, but rather generalists adapted to a wide variety of habitats.

## Methods

### Sampling sites

**Fig. 2:**
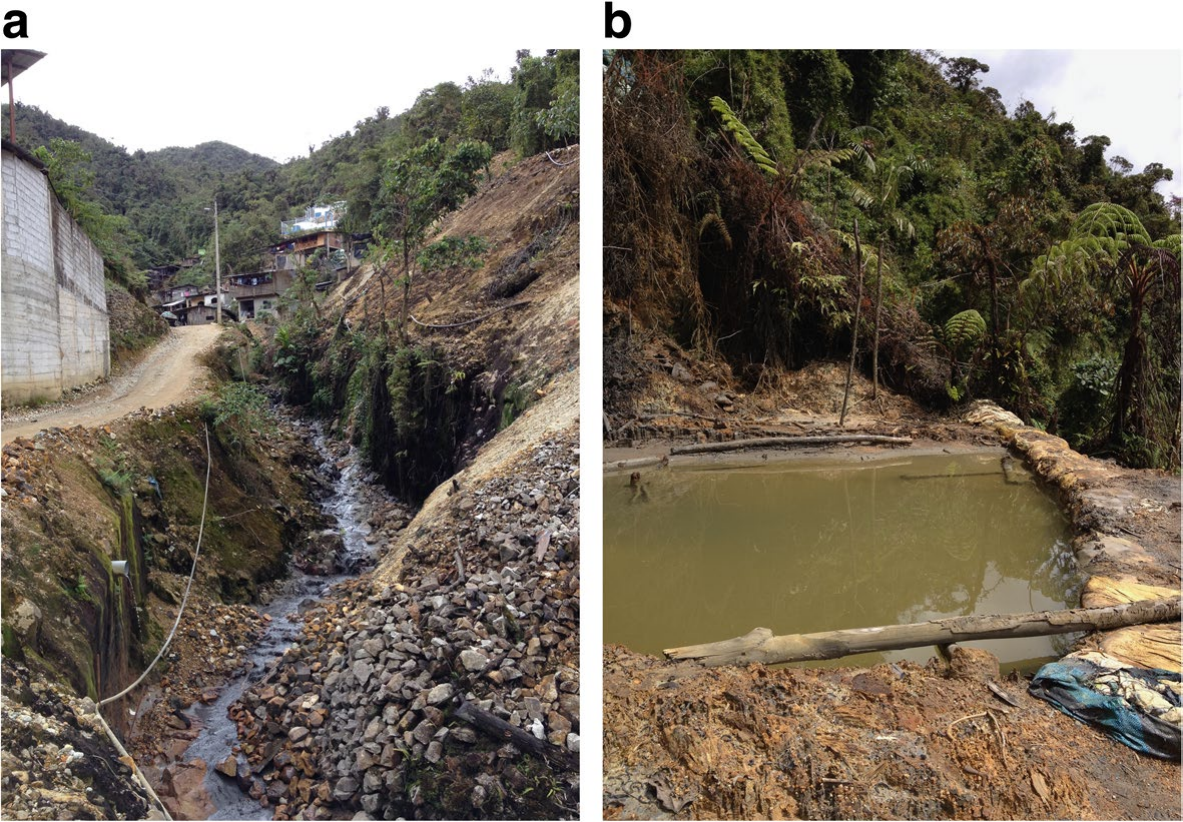
Sampling sites located in Zamora-Chinchipe Province were soil and root samples were collected: wastewater canal at Chinapintza site (**a**) and waste collection pool at La Pangui site (**b**). Both photographs were taken by JPS

The study area is located in Zamora-Chinchipe Province, beside the Condor mountain range, southeast Ecuador close to the Peruvian border. The study area comprises two sites, the first one located at Chinapintza locality (1854 m a.s.l. 04º02′19.74’’S, 78º36′27.35’’W) where a large volume of ASGM activities are being carried out. The mineral richness of Chinapintza was rediscovered and exploited in a disorderly manner through an artisanal mining process in the early 1980s, and since 1993 has been additionally subject to extensive exploitation, with more than 22,580 m of drilling by Chinapintza Gold Project, a small-scale gold mining operation [36]. Samples were collected from a nearby wastewater canal (Fig. 2a).

The second site is La Pangui (1677 m a.s.l., 04º02′56.29’’S, 78º34′58.59’’W) located about 5 km from Chinapintza. The intensity of ASGM activities is lower than in Chinapintza. Samples were collected close to a waste collection pool (Fig. 2b). The weather in Chinapintza is typical for areas situated at this elevation along the Amazonian side of the Andes, with average daily temperatures ranging between 18 – 29ºC and relative air humidity between 80 – 85%. Annual rainfall ranges between 2000 and 4000 mm with increase in rainfall between February and April [11]. The two sampling sites share the same climatic characteristics due to their proximity.

### Sampling of plant roots and soil

Sampling was conducted between October 2014 and July 2015. Plants and root samples were collected from herbs, trees, and shrubs common to both polluted locations. Fine roots (diameter < 0.2 cm) were collected by identifying the main root and carefully following the secondary roots until the fine roots were located to evaluate mycorrhizae. At Chinapintza, roots of 1 to 2 individuals of the plant species *Axonopus compressus* (SW.) P. Beauv (Poaceae), *Erato polymnioides* DC. (Asteraceae)*, Miconia zamorensis* Gleason (Melastomataceae) and *Medinilla* sp. (Melastomataceae) were collected, in total 9 samples. In La Pangui roots of 2 to 7 individuals of the plant species *E. polymnioides*, *Medinilla* sp., *M. zamorensis*, *Colocasia* sp. (Araceae), and *Cyathea* sp. (Cyatheaceae) were collected, in total 21 samples. Together, 30 samples. Plant species identity was determined based on existing collections at Herbarium of the Universidad Técnica Particular de Loja (HUTPL) and Chamba et al. [11]. Collected specimens correspond to *Erato polymnioides* HUTPL11521, *Axonopus compressus* HUTPL 12,456 and *Miconia zamorensis* Chicago Nat. Hist. Museum 1,188,658.

Only small plants were present at each site due to the harsh conditions (Fig. 2 a, b). Samples were packed in bags and transported to the laboratory. Approximately 1 kg of root zone soil was collected per each sampled plant at a depth between 0 and 20 cm. The soil samples were air-dried and preserved at room temperature until soil analyses. In addition, fine roots were selected and cleaned with tap water, labeled, stored in 70% ethanol, and kept for subsequent analysis. The research permit was issued by Ministerio del Ambiente del Ecuador (MAE-DNB-CM-2015–0016).

### Analysis of physico-chemical parameters and heavy metals from soils

The soil samples were sent to the Laboratorio de Manejo de Suelos y Aguas at Instituto Nacional de Investigaciones Agropecuarias (INIAP), Quito, Ecuador, for analysis. Physical properties such as pH, and chemical ions like ammonia, chloride, calcium, magnesium, sodium, potassium, ferrous, boron ions were tested. The analyses were performed according to standard methods [6, 25].

The soil samples were analyzed individually, 1 g of the homogenized oven dried soil was subjected to digestion with a mixture of HCl and HNO_3_ in a 3:1 ratio (v/v). Samples were left for one week to soak in the acid, after which they were digested in an open thermal block (Environmental express 54 Hot block SC154) for 2 h. After cooling, the samples were diluted up to 100 ml with HCl 0.1 M and stored until metal analyses. Prior to measurements, the solutions were filtered through filter paper. The concentrations of heavy metals in digested solutions were analyzed immediately using a flame atomic absorption spectrophotometry (FAAS). The metals evaluated were Cd, Pb, Al, Cu, Zn, Cr, Au and Hg.

### DNA isolation, PCR and molecular cloning

Colonization of the ethanol fixed mycorrhizae was examined using a standard staining method [17] to select root fragments for DNA isolation. Ten to fifteen root fragments of 1 cm per each plant were used for total DNA extraction using the DNeasy Plant Mini Kit (Qiagen, Hilden, Germany), according to the manufacturer’s protocol. The 18S nrDNA was amplified by two rounds of PCR. The first PCR was performed with primer pair NS1/NS4 (5’-GTA GTC ATA TGC TTG TCT C-3’ and 5’-CTT CCG TCA ATT CCT TTA AG-3’, [57] and for the nested PCR (second PCR) using the Glomeromycota-specific primer combination AML1 (5’-ATC AAC TTT CGA TGG TAG GAT AGA-3’, [32] and AML2 (5’-GAA CCC AAA CAC TTT GGT TTC C-3’, [32]. The reaction volume was 25 µl using the Phusion High-Fidelity PCR Mastermix (Finnzymes, Espoo, Finland), 200 mM for each dNTP (Life Technologies, Eggenstein, Germany), 0.5 mM for each primer (Biomers, Ulm, Germany) and 0.2 mL 1% Bovine Serum Albumin (BSA; Sigma). The PCR conditions were as follows: 3 min initial denaturation at 94 °C, followed by 30 cycles of 1 min denaturation at 94 °C, 1 min primer annealing at 50 °C and 1 min extension at 72 °C, followed by a final extension period of 10 min at 72 °C [57].

The success of PCR amplification products was tested in 1% agarose gel stained with GelRed™ Safe Nucleic Acid Gel Stain (Biotium, Hayward, USA), the expected fragment size of amplicons was approximately 0.8 Kb. The amplicons were cloned using the Zero Blunt® TOPO® PCR Cloning Kit (Invitrogen), according to the manufacturer’s protocol. Twelve colonies per individual were selected for PCR amplification using modified M13F and M13R primers [30]. The cloned mycorrhizal DNA was purified using S.N.A.P Miniprep purification kit (Invitrogen), using the manufacturer’s instructions. Clones were sequenced by Macrogen (Seoul, Korea) using universal primers M13F and M13R.

### Molecular phylogeny and OTU calculation

Raw sequences obtained from samples of Chinapintza and La Pangui were edited with Sequencer software (Version 4.9, Gene Codes, Ann Arbor, Michigan). Consensus were generated, and with the resulting consensus sequences, a BLAST search was performed against the nucleotide sequence database (NCBI, [5] and MaarjAM database [38]. Sequences from the databases with high similarity to our sequences were added to the dataset to obtain the final alignment.

Operational Taxonomic Units (OTUs) were defined as surrogates for species on the basis of sequence similarity with OPTSIL [18]. A first analysis of OTUs was performed with sequences from heavy metal polluted sites using a cut-off value of 99% similarity for the about 800 bases long section of the 18S nrDNA. The linkage fraction was 0.5, which combines two clusters if the distances between 50% of the sequences in each cluster are equal or below the cut-off value [18]. Later, a new OPTSIL analysis was done with OTUs obtained in the first analysis and including the downloaded sequence(s) from NCBI and MaarjAM, singleton sequences were removed.

Sequence alignments of the complete matrix were done with MAFFT v6.847b (http://mafft.cbrc.jp/alignment/software/; strategy G-INS-i, [27]. A maximum likelihood (ML) analysis was performed in MEGA 5 [53], under the General Time Reversible DNA substitution model with 1000 bootstrap replicates. The resulting tree was edited using FigTree Ver. 1.4.3 [40]. Calculated OTUs from the heavy metal polluted soils were drawn in this ML tree. Finally, 64 sequences were deposited in GenBank with accession numbers OL652886–OL652949.

## Data Availability

The DNA sequences generated in this study are available in the NCBI GenBank (https://www.ncbi.nlm.nih.gov) under the accession numbers OL652886–OL652949 and 18S nrDNA alignment data is available at BioProject SUB12460283.

## Abbreviations

HMM: Heavy metals and metalloids
AMF: Arbuscular mycorrhizal fungi
ASGM: Artisanal and small**-**scale gold mining
BSA: Bovine Serum Albumin
OTUs: Operational Taxonomic Units
